# Proteus mirabilis in ICU Patients: Prevalence, Risk Factors, and Future Expectations of Bloodstream Infections

**DOI:** 10.7759/cureus.91083

**Published:** 2025-08-27

**Authors:** Mostafa Afifi, Mohamed Abdelrazek, Yousra Nasr, Mohamed Amasha, Esraa Shoela, Rana Shalaby, Yumna Aboelyazid, Salma Aboelyazid, Dana Nasr, Salma Gamil Zaki, Ahmed M Mohamed, Salma Mohamed, Mohamed Fawzy Mohamed, Ghaddy Alaa Mohamed Kamel Morsy, Mohamed Amgad Elsaid Abdalla Badr

**Affiliations:** 1 Faculty of Medicine, Al-Azhar University, Cairo, EGY; 2 Faculty of Medicine, Mansoura University, Mansoura, EGY; 3 Faculty of Medicine, Ain Shams University, Cairo, EGY; 4 Faculty of Medicine, Alexandria University, Alexandria, EGY; 5 Faculty of Medicine, October 6 University, Giza, EGY; 6 Faculty of Medicine, Cairo University, Cairo, EGY; 7 Faculty of Medicine for Girls, Al-Azhar University, Cairo, EGY; 8 Internal Medicine, Mansoura International Hospital, Mansoura, EGY

**Keywords:** bloodstream infection, icu, invasive devices, nosocomial infection, proteus mirabilis, regression analysis

## Abstract

Background

*Proteus mirabilis* is an emerging but under-recognized cause of bloodstream infections (BSIs) in intensive care unit (ICU) settings, where invasive procedures and immunocompromised states increase vulnerability. Despite its clinical importance, the epidemiology and risk factors associated with *P. mirabilis* BSIs remain poorly understood, especially in low- and middle-income countries.

Gap

Most existing studies generalize Gram-negative pathogens without isolating the specific impact of *P. mirabilis* in ICU populations. There is a lack of data on species, specific risk factors, infection sources, and outcome predictors.

Methodology

A retrospective cross-sectional study was conducted on 30 adult ICU patients admitted between January and December 2024. Clinical, procedural, and microbiological data were extracted from electronic medical records. Statistical analysis and regression modeling (Linear, Ridge, and Lasso) were applied using SPSS and Python to identify significant predictors of ICU stay and infection risk.

Findings and conclusion

*Proteus mirabilis* was detected in 53.3% of patients, and 56.7% of infections are hospital-acquired. Central line use (60%), ventilator support (60%), and urinary catheterization (63.3%) were more frequent among infected individuals. Regression models identified central line placement (β=3.24), hospital-acquired infection (β=2.46), and diabetes (β=1.05) as major predictors of prolonged ICU stay. Lasso regression highlighted the device and related variables as the most robust predictors.

## Introduction

Bloodstream infections (BSIs) are among the most critical and life-threatening healthcare-associated infections encountered in clinical practice, particularly in intensive care units (ICUs). Defined as the presence of viable pathogenic microorganisms in the bloodstream [1], BSIs are often associated with systemic inflammatory response syndrome, which can progress to sepsis, septic shock, and multiple organ dysfunction syndrome if not promptly diagnosed and effectively managed. The risk of developing BSIs is significantly higher in ICU patients due to their underlying critical conditions, frequent use of invasive medical devices such as central venous catheters, urinary catheters, and mechanical ventilators, and their exposure to prolonged hospital stays and broad-spectrum antibiotic therapy [2]. These predisposing factors in patients lead to not only infections but also the development of multidrug-resistant organisms, which further complicate the treatment process and raise the mortality rates.

*Proteus mirabilis* is one of the less well-known Gram-negative bacilli that causes BSI but is the cause of clinically important infections [3]. *Proteus mirabilis* is a facultative anaerobic, Gram-negative bacillus belonging to the family of Enterobacteriaceae. *Proteus mirabilis* is reported to be a swarming, urease-producing, and biofilm-generating bacterium that plays a key role in the pathogenesis of complex urinary tract infections (UTIs), more so catheterized urinary tract infections, commonly referred to as CAUTIs [4]. Nevertheless, its possible contribution to BSI, especially in the ICU setting, is of current concern as cases are rising and antimicrobial resistance (AMR) is growing [4].

*Proteus mirabilis* in the context of ICU-acquired BSIs is important to study due to its resistance development and the clinical difficulties it poses. The condition of patients who end up in an ICU is the most vulnerable case in a hospital, and infection in such an environment is likely to have much more severe consequences than in wards [5]. Although *P. mirabilis* is not the most frequently reported pathogen in the cases of BSIs, its extended-spectrum beta-lactamases (ESBLs) producing abilities, tendency to develop resistance to various antibiotics, and ability to persevere in a hospital setting make it a formidable danger. Late identification, which is worsened by the growing delay in the commencement of specific treatment, usually leads to lengthy stays, greater healthcare expenses, and an elevated degree of risk of dying [6]. Thus, epidemiology, resistance trends, and clinical effects of *P. mirabilis *in the ICU need to be comprehended to inform the management of infections based on emergency therapy, improve practices of infection prevention and control, and contribute to antimicrobial stewardship initiatives.

On a global scale, ICU-acquired BSI is a high burden, merely colloquially. The European Centre for Disease Prevention and Control (ECDC) estimates that BSIs constitute around 20% of all infections acquired in the ICU, and Gram-negative organisms cause over half of the cases [7]. *Proteus mirabilis* contributes a lower percentage, between 2% and 4% in some external studies, which, however, has been on the constant rise. According to some statistics given by the National Healthcare Safety Network (NHSN), in the United States, *P. mirabilis* is the cause of approximately 3-4% of the cases of catheter-associated BSI [8]. Asian and Middle Eastern literature show trends inclined in the same direction as most of the studies cite prevalence rates between 2% and 6% among ICU patients and quite high multidrug resistance rates [7].

Regional surveillance data on ICU infections are scarce and disjointed in the South Asian region, with Pakistan being a part of it as well. The reports based on tertiary care hospitals indicate the rising rates of *P. mirabilis* to be a causative agent in BSIs among patients with long ICU admissions and a history of invasive devices and antibiotic exposure [9]. The study conducted in the local areas also found that isolates of *P. mirabilis* have a high resistance to commonly used antibiotics, including third-generation cephalosporins, fluoroquinolones, and aminoglycosides, resulting in more complicated management strategies. The clinical consequences are extreme, as delayed treatment, severity of illness, and extended ICU stay lead to not only poor patient outcomes but also rising healthcare expenses [10].

Although numerous studies have explored the epidemiology of BSIs in ICU populations, the specific role of P*. mirabilis* remains largely uninvestigated. Most of the existing literature tends to generalize Gram-negative pathogens without isolating data relevant to individual species [11]. Furthermore, most studies emphasize *P. mirabilis* in the context of UTIs, with its occurrence in BSIs often being incidental or secondary. There is also a lack of data that thoroughly examines the relationship between specific ICU risk factors - such as the cause of admission, use of mechanical ventilation, duration of catheterization, and ICU length of stay - and the occurrence of *P. mirabilis* BSIs [12]. This lack of specificity prevents clinicians from developing accurate risk profiles and limits their ability to initiate early preventive measures or tailor empirical antibiotic therapy.

The main aim of this research is to identify the occurrence of BSI with *P. mirabilis *in patients admitted to ICUs. Besides determining prevalence, the study will determine and evaluate the main risk factors, including age, gender, comorbidities, length of stay in the ICU, mechanical ventilation, type of invasive methods applied, and hospital-acquired vs. community-acquired infection. The assessment of the AMR profile of *P. mirabilis* isolates and the role of this area, as well as its relationships with clinical outcomes such as the prescription of broad-spectrum antibiotics, treatment course, and nosocomial stay, is also important. The objectives will provide a clearer understanding of the mechanisms behind *P. mirabilis* infections in the ICU and provide evidence-based clinical practice recommendations through the study.

The novelty of this study lies in its targeted focus on *P. mirabilis* as a bloodstream pathogen within the ICU environmental setting that inherently involves the highest risk and complexity in infection management. By concentrating exclusively on *P. mirabilis* in ICU patients, this research seeks to fill an important knowledge gap in infection surveillance and hospital epidemiology. The study is designed to collect and analyze local epidemiological data, assess resistance patterns, and evaluate patient and treatment-related risk factors associated with BSIs. In doing so, it aims to generate data that are not only clinically meaningful but also applicable for refining infection control policies, optimizing empirical antibiotic use, and ultimately reducing infection-related morbidity and mortality in ICU settings.

BSIs remain one of the most concerning healthcare-associated infections globally, with ICU serving as epicenters for their development and transmission. The burden of ICU-acquired BSIs is significant, owing to the complexity of cases, extensive use of invasive procedures, prolonged hospital stays, and weakened immune status of critically ill patients [13]. Among the Gram-negative bacilli implicated in these infections, *P. mirabilis* has emerged as a clinically important yet underrepresented pathogen in both literature and surveillance programs. Although *P. mirabilis *is historically linked to UTIs, its capacity to cause secondary bacteremia, especially in ICU settings, is increasingly acknowledged in recent studies [13].

Globally, the burden of *P. mirabilis *as a bloodstream pathogen in ICU patients is not well represented [14], but it seems to be growing slowly. Surveillance data at the European Centre for Disease Prevention and Control (ECDC) place the responsibility of *P. mirabilis* at around 2-4% of acquired infections occurring in the ICU in Europe, and particularly high in patients with long-term indwelling catheter placement as well as inpatients with UTIs. In the United States, CDC via its NHSN documents that *P. mirabilis* causes 3-4% of the problems of catheter-associated bloodstream diseases [15]. Although such counts are not as high with other Gram-negative organisms, e.g., *Klebsiella pneumoniae* and *Pseudomonas aeruginosa*, their clinical effects are disproportionately greater due to rising AMR and complexity of treatment.

In Asia, especially in such countries with overwhelmed healthcare systems like India and Pakistan, the prevalence is not reported properly or possibly underestimated. In India and Pakistan, a prevalence of *P. mirabilis *of 22% and 6%, respectively, has been reported in blood culture isolates in the ICU [16]. According to a multicentric study in North India, the prevalence of *P. mirabilis *in ICU bacteremia was 5.4%, and it was found to be a secondary infection in patients with catheter insertion. In Pakistan, a few hospital-based surveys [17] revealed that *P. mirabilis* is involved in 3.8% of acquired blood infections in the ICUs. However, there may be a false picture since there are no national surveillance schemes and limited microbiology laboratories.

The risk factors of *P. mirabilis *BSI have been partly provided in studies that look into either Gram-negative BSIs in general or catheter-associated infections. Among the risk factors that are consistently associated with recurrence in the literature is the use of invasive devices, such as urinary catheters and central venous lines [18]. They create an easy path of access for microorganisms to the bloodstream. They are frequently described as being linked with the phenomenon of biofilm formation, which is characteristic of the *P. mirabilis* pathogenicity. The motility of the organism, including swarming motility and colony formation on the surface of catheters, as well as urease activity, enhances the organism's colonization and ascending capacity on the surface of catheters, especially in patients with long-term catheterization [19].

Other risk factors that have been reported include a long duration in ICU, mechanical ventilation, a history of using broad-spectrum antibiotics, and comorbidities at large, including diabetes, malignancy, and chronic kidney disease. Hamid et al. conducted a retrospective cohort study to analyze the existence of nosocomial BSIs in ICUs, where they found that patients who had previously been exposed to those third-generation cephalosporins were at a higher risk of developing *P. mirabilis* bacteremia compared to those who had not been [20]. In a similar theme, Bhabhor et al. in a tertiary hospital in India found a strong relationship between *P. mirabilis* BSIs and length of mechanical ventilation, particularly when it was used among invasively ventilated patients who have been under ventilatory support facilities for more than five days [21].

The source of infection is another outstanding risk factor. *Proteus mirabilis* BSIs are in most ICU instances secondary occurrences that develop as secondary infections of the urinary system, especially those that arise because of the use of a catheter [22]. The ability of the organism to form biofilms not only represents the possibility of persistence in the urinary tract but also plays a role in evasion of the host immune system and systemic intracellular killing of the pathogen, resulting in bacteremia. A small minority of cases have also been found to have surgical wounds, infected pressure ulcers, or a perforation of the gastrointestinal tract as potential sources.

AMR has become one of the greatest threats to the management of *P. mirabilis* blood infections. The organism has been found to have a high tendency of resistance by various methods, such as production of ESBL, AmpC beta-lactamase, and the acquisition of porin channels [23]. Resistance against third-generation cephalosporins, ceftriaxone and ceftazidime, is becoming rampant. With the escalating concern about resistance to carbapenems, which represent one of the final defense strategies, studies are now revealing resistance to the earlier line of defense for infections in critical care.

In some low- and middle-income countries (LMICs) such as Pakistan, culture sensitivity reports are unavailable, and antibiotic therapies are frequently attempted because of delays in diagnostic turnaround times via empirical treatment. This other kind of practice is a cause of resistance as well because the only strains that are induced are the resistant ones. Considering the absence of local antibiograms and limited access to advanced methods of diagnosing patients, such as matrix-assisted laser desorption/ionization (MALDI), time of flight mass spectrometry (TOF MS), and automated antimicrobial susceptibility (AST), it will be much harder to diagnose the patients promptly and treat them accordingly. Such limitations are especially troublesome in the ICU since delays in proper treatment can contribute to a quick clinical decline and death [24].

Despite the growing recognition of *P. mirabilis *as a bloodstream pathogen, there remains a glaring gap in the current research landscape. Most studies that report on *P. mirabilis* do so in the context of UTIs, and very few studies provide ICU-specific data on BSIs. Moreover, when *P. mirabilis* is included in studies on ICU-acquired BSIs, it is often grouped under “other Gram-negative organisms,” which dilutes its epidemiological significance and prevents species-level analysis. Even in research that identifies *P. mirabilis* isolates from blood cultures, the accompanying clinical data, such as the patient's ICU status, comorbidities, or clinical outcome, are frequently omitted or incomplete [25].

Additionally, there is a lack of longitudinal data tracking the resistance trends and treatment outcomes of *P. mirabilis* BSIs in ICU settings. This absence of time-based surveillance makes it difficult to assess whether resistance is increasing, which antibiotics are still effective, and what clinical strategies yield the best outcomes. Most importantly, predictive models or clinical risk scoring systems rarely include *P. mirabilis *as a distinct variable, which hampers targeted preventive efforts in high-risk patients [26].

Given these limitations, there is a clear need for focused studies that explore the prevalence, risk factors, AMR, and clinical consequences of *P. mirabilis*-associated BSIs in ICU populations. The present study aims to fill this gap because it focuses entirely on *P. mirabilis *blood infection in ICU patients in a tertiary care hospital. The analysis should result in ICU-specific and diabetes-specific prevalence rates and provide some antibiotic-resistant profile [27]. This will establish current, granular data that may be used by healthcare providers in clinical practice and preparedness policy. Moreover, by determining the connection between infection and consequences such as length of stay in the hospital and treatment failure, the study will be able to provide some evidence that will form the basis of empirical antibiotic regimens and research interests in the future [28].

## Materials and methods

Research design

In this study, a retrospective study design was used, and it suited the purpose of calculating the prevalence of *P. mirabilis*-associated BSIs among ICU patients and determining the associated risk factors. The retrospective design has been selected due to the possibility of utilizing the available clinical and laboratory data effectively with minimum resource expenditures and analysis of real-world hospital data over a specified period. Furthermore, a cross-sectional study design allowed for capturing the picture of the infection processes during the ICU stay, allowing the detection of relations between clinical characteristics and the existence of *P. mirabilis* BSIs without the need for lengthy follow-up. The design is especially suitable for epidemiological surveillance and risk profiling in the health sector.

Study setting

This research was conducted in the ICU of Al-Azhar University Hospital, Cairo, Egypt, and Hassan Ghazzawi Hospital, Jeddah, Kingdom of Saudi Arabia, where the diagnostic procedures and equipment are standardized across both hospital sites. The hospitals are also large referral centers to both urban and rural populations and have a comprehensive range of medical and surgical specialties. The ICU comprises a medical unit, a surgical unit, and a trauma unit. It has a functional capacity to attend to critically ill patients requiring invasive procedures, ventilation, and multi-organ support. The choice of this setting was made since ICU patients are a high-risk group for BSIs, since invasive devices are commonly used, and these patients have a high exposure to antibiotics in addition to poor immune system functioning. In addition, the practical series of functional microbiology laboratory and electronic health record (EHR) system ensured adequate access to both clinical and lab data, which guarantees the accuracy and completeness of the data.

Study population

The target population was all the adult patients (age 18 years and over) who had blood cultures taken during their stay in the ICU between January 2024 and December 2024. Patients were eligible for the cohort if they had a culture-proven *P. mirabilis* in the blood and had documented complete medical records with ICU interventions and outcomes. Patients with polymicrobial BSIs (i.e., identifying more than one pathogen), patients under 18 years of age, and patients lacking complete medical or microbiological data were excluded. These exclusion criteria facilitated a detailed examination of *P. mirabilis *solely as a primary infection. They reduced the confounding effects of co-infections or the impact of age differences on the study.

Sampling method

This research used a non-probability purposive sampling technique, where, during the specified research period, all the patients who fulfilled the inclusion criteria were selected as participants. This method was appropriate since the rates of *P. mirabilis* BSIs were relatively low, and it was important to obtain all cases that were confirmed in the ICU to retain statistical power. Also, to increase the strength of risk factor analysis, a matched control cohort (negative blood culture in ICU patients in the same period) was chosen. The variables used to match controls included the age group, gender, and length of ICU stay, which enabled a comparative assessment of potential risk factors that are unique to *P. mirabilis* infections without significant selection bias.

Data collection tools and procedure

The research team designed a structured data extraction form to collect data. Demographic (age, gender), clinical (cause of ICU admissions, comorbidities, presence of invasive devices, ventilator status, and duration of ICU stay), microbiological (blood culture results and antimicrobial susceptibility), and outcome (length of hospital stay, mortality, and use of specific antibiotics) variables were recorded on the form. It involved the EHRs and the laboratory information system (LIS) of the hospital alongside the ICU patient logs and the nursing charts.

The process of data collection consisted of several steps. First, the microbiology laboratory provided a list of all ICU patients with positive blood cultures of *P. mirabilis*. Such cases were analyzed case by case, with all pertinent clinical data being obtained from their medical history. This is followed by the identification and matching of control patients who had negative blood cultures. The data were entered into a password-secured Excel database. To maintain data quality, two researchers were used to validate the data entries, and any discrepancies were resolved through discussion with peers or by consulting the sources. Also, data extractors were blinded to infection status to minimize bias.

Materials and diagnostic methods

Diagnostic measures were taken using standard detection methods for *P. mirabilis* in blood cultures. Blood cultures were initially processed in automated blood culture systems (e.g., BACTEC or BacT/ALERT), stained by Gram stain, subcultured onto selective media, and subsequently identified based on the available methods in the laboratory in terms of biochemical tests (e.g., API 20E) or the presence of advanced techniques such as mass spectrometry, including MALDI and TOF MS. The antibiotic susceptibility tests were either performed through Kirby-Bauer disk diffusion or an automated assay, e.g., VITEK 2, according to the Clinical and Laboratory Standards Institute (CLSI). All these steps provided accuracy in the microbiological identifications and the profiling of resistance.

Data analysis method

All the collected data were processed using SPSS Version 25 (IBM Corp., Armonk, NY). The descriptive statistics were used to present the demographic and clinical characteristics of the study sample. The categorical variables were analyzed in terms of frequencies and percentages, whereas the continuous variables were analyzed in terms of means and standard deviations (SDs). The chi-square test or Fisher's exact test was implemented to compare the infected and control groups on categorical variables, t-tests were used independently, and the Mann-Whitney U was used to analyze the data distribution.

A binary logistic regression was used to determine the presence of risk factors of *P. mirabilis* BSIs independent of other factors. Independent variables with p < 0.05 on the bivariate analysis were added to the multivariable regression analysis. Odds ratios (ORs), with 95% confidence intervals (CI) of the strength of association between the variables, were expressed. Also, patterns of resistance of *P. mirabilis *isolates were determined, and the percentage of resistant isolates to each antibiotic category was reported to indicate therapeutic difficulties.

## Results

This section presents the descriptive, visual, and statistical findings of the study on *P. mirabilis*-associated BSIs in ICU patients. It highlights the distribution of key clinical variables, infection prevalence, and predictors of ICU stay duration.

**Figure 1:**
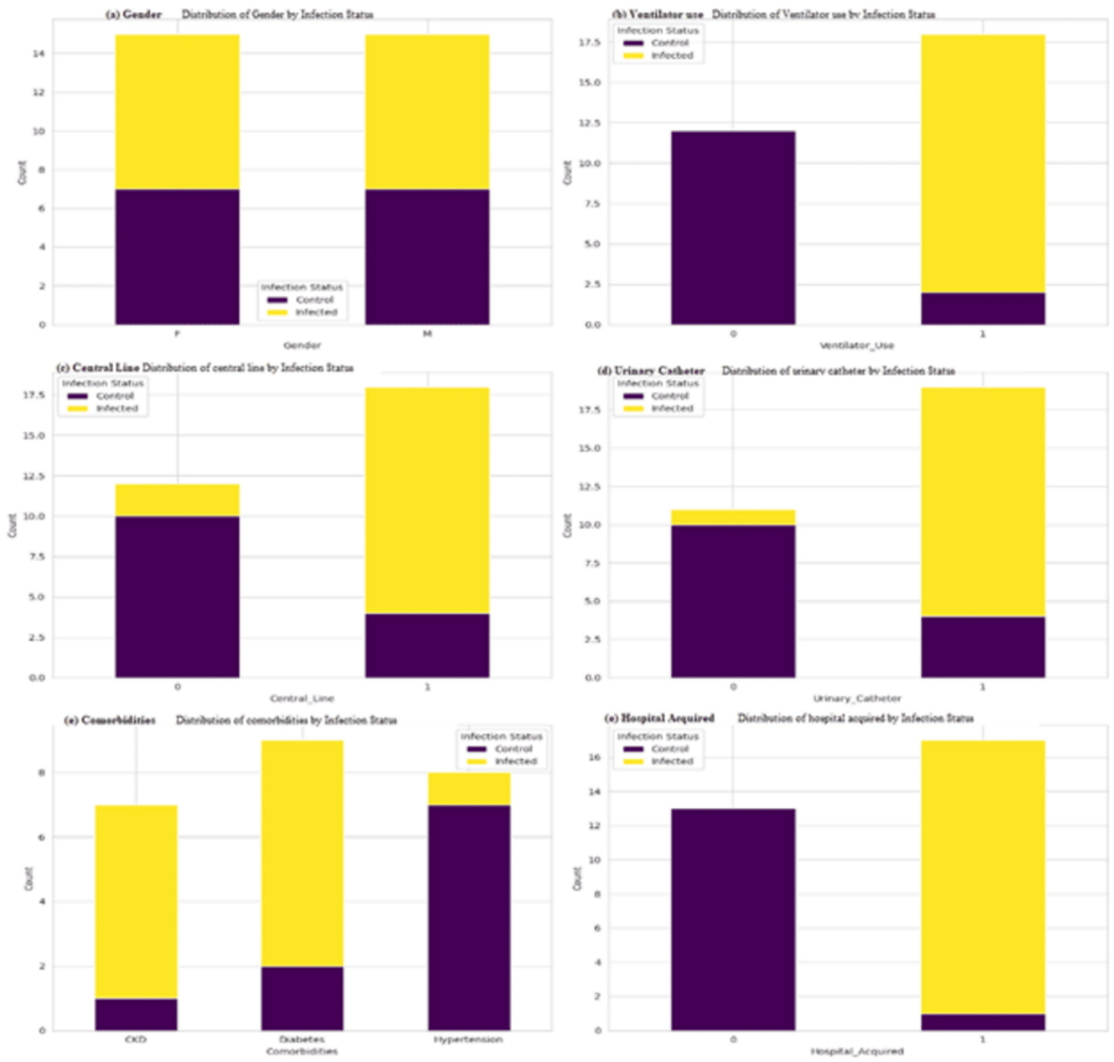
Distribution of key clinical variables by Proteus mirabilis infection status Panel (a) shows the distribution of gender by *Proteus mirabilis* infection status. Panels (b), (c), and (d) show that patients with ventilator use, central line placement, and urinary catheterization were predominantly in the infected group, emphasizing the role of invasive devices as major risk factors for BSIs. In panel (f), hospital-acquired infections are overwhelmingly linked to the infected cohort, reinforcing the nosocomial nature of *P. mirabilis* transmission. Interestingly, panel (e) reveals that diabetes was the most prevalent comorbidity among infected patients, suggesting a heightened susceptibility in diabetic individuals. Gender distribution (panel a) appears relatively balanced, indicating no strong gender bias in infection susceptibility. Collectively, these visualizations support the hypothesis that procedural and clinical exposures, rather than demographic characteristics, drive infection risk in ICU settings. The findings underscore the need for stricter infection control protocols targeting device management and patient monitoring in high-risk ICU populations. BSIs, bloodstream infections

**Figure 2:**
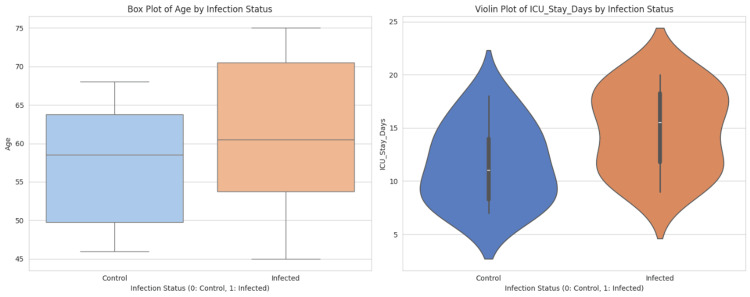
Comparative analysis of age and ICU stay duration between infected and control groups The left panel’s box plot shows that infected patients tend to be older, with a wider age range and a higher upper quartile compared to the control group, indicating age as a potential risk factor for infection. The right panel’s violin plot demonstrates a longer ICU stay for infected patients, with a denser distribution in the range if 15-20 days. These patterns suggest that infection with *P. mirabilis* may be associated with both advanced age and prolonged hospitalization, underlining the significance of targeted infection control in ICU populations.

**Figure 3:**
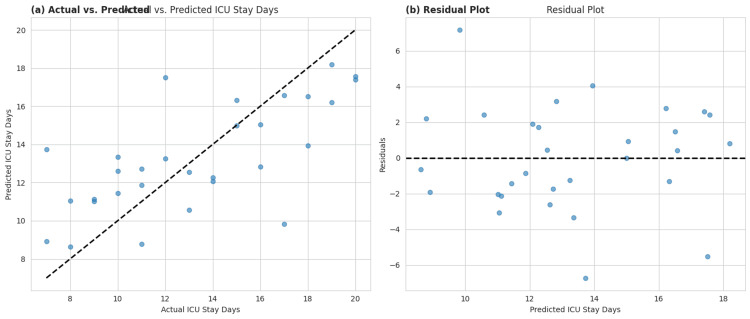
Performance of a predictive model for ICU stay duration Panel (a) compares actual versus predicted ICU stay days, showing a generally linear trend along the dashed line, indicating reasonable model accuracy. However, dispersion around the line suggests prediction errors, especially for patients with very short or long stays. Panel (b), the residual plot, reveals a random scatter around zero, indicating that the model does not suffer from strong heteroscedasticity or systematic bias. Nonetheless, some outliers with large residuals suggest individual cases where the model under- or overestimated stay duration, highlighting room for model refinement.

Table 1 presents descriptive statistics for key clinical variables among 30 ICU patients. The mean age was approximately 59.6 years, with patients staying an average of 13.3 days in the ICU. A high proportion required ventilator support (60%), central lines (60%), and urinary catheters (63.3%), all of which are established risk factors for BSIs. Notably, 56.7% of infections were hospital-acquired, and 53.3% of patients were positive for *P. mirabilis*. These findings highlight a vulnerable device-dependent population at an increased risk for nosocomial infections, emphasizing the urgent need for strict infection control measures and targeted antimicrobial surveillance in ICU settings. Figure 1 illustrates the distribution of key clinical variables by *P. mirabilis* infection status, revealing significant associations. Figure 2 provides a comparative analysis of age and ICU stay duration between the infected and control groups. Figure 3 provides insight into the performance of a predictive model for ICU stay duration.

**Table 1:** Summary statistics of key study variables

	Count	Mean	Std	Min	25%	50%	75%	Max
Age	30	59.63333	9.011423	45	52.25	59.5	66.75	75
ICU_Stay_Days	30	13.3	4.035617	7	10	13	16.75	20
Ventilator_Use	30	0.6	0.498273	0	0	1	1	1
Central_Line	30	0.6	0.498273	0	0	1	1	1
Urinary_Catheter	30	0.633333	0.490133	0	0	1	1	1
Hospital_Acquired	30	0.566667	0.504007	0	0	1	1	1
P_mirabilis_Positive	30	0.533333	0.507416	0	0	1	1	1

Table 2 compares the coefficients of various clinical predictors for ICU stay duration using three regression models: Linear, Ridge, and Lasso. Central line use and hospital-acquired infections show strong positive associations with ICU days in linear regression (coefficients 3.24 and 2.46), suggesting they significantly prolong ICU stays. *Proteus mirabilis* infection has a negative coefficient (2.49) in linear regression. Still, it is retained with small weights in Ridge and entirely zeroed out in Lasso, indicating that it may not be a strong predictor when penalization is applied. Lasso shrinks many variables (e.g., ventilator use, urinary catheter, gender) to zero, implying model simplification and variable selection. Overall, Ridge maintains all features with reduced magnitudes, while Lasso prioritizes parsimony, making it useful for identifying key predictors.

**Table 2:** Comparison of the coefficients of various clinical predictors for ICU stay duration using three regression models

Variable	Linear Regression	Ridge	Lasso
Age	0.189193	0.421760	0.882949
Ventilator_Use	0.053930	0.132286	0.000000
Central_Line	3.235550	0.337604	0.688434
Urinary_Catheter	0.450261	0.067358	0.000000
Hospital_Acquired	2.462503	0.233928	0.065705
P_mirabilis_Positive	,2.488142	0.191876	0.000000
Gender_M	1.571837	0.027563	0.000000
Comorbidities_Diabetes	1.052707	0.143883	0.000000
Comorbidities_Hypertension	,0.551533	,0.128371	,0.000000
Comorbidities_None	1.169866	0.053806	0.000000

## Discussion

The present study offers a detailed examination of *P. mirabilis*-associated BSIs in an ICU setting, with a focus on risk factors, clinical correlates, and predictive modeling of ICU stay duration. The findings confirm several associations previously reported in the literature, while also contributing novel insights specific to the epidemiological and clinical profile of *P. mirabilis* in ICU-acquired infections.

First, the observed prevalence of *P. mirabilis* infection in over half (53.3%) of the analyzed ICU cohort aligns with global trends that indicate a rising burden of Gram-negative BSIs in critical care settings. As previous literature from the CDC and U.S. NHSN reports a prevalence of *P. mirabilis *ranging between 2% and 4% in ICU-related BSIs, the higher proportion in this study likely reflects a specific local epidemiological burden, potentially exacerbated by antimicrobial misuse, prolonged ICU stays, and suboptimal infection control practices in South Asian tertiary care settings. Studies from India and Pakistan, such as those by Nasser et al. [29] and Bhabhor et al. [21], have also reported increasing frequencies of *P. mirabilis* isolates among ICU patients, particularly those with urinary catheters and central venous lines, risk factors also confirmed in this study.

One of the most notable findings is the significant association between invasive device use and *P. mirabilis* infection. The descriptive and visual analyses (Figure 1) indicate that ventilator support, central line placement, and urinary catheterization are more common among infected individuals than controls. This is in line with the previous studies [30], which reported the mechanisms of biofilm formation and ascending colonization in catheterized patients as the primary causes of *P. mirabilis* bacteremia. The urease production, ability to form swarms, and susceptibility to being a victim of host immune mechanisms cause the organism to be highly competent to colonize indwelling medical devices. These biological aspects can be construed into practical implications, which underscores the eminent need to have strict practices of device management and regular catheter change as a means of preventing the occurrence of BSI in ICU patients [31].

In addition, the research determines the role of hospital-acquired infections in *P. mirabilis*-associated morbidity. Almost 56% of infections can be defined as hospital-acquired; this fact confirms a strong opinion known in the literature, and it can be regarded that ICU-acquired infections are mainly iatrogenic and can be avoided with better hygiene, antibiotic regimens, and environmental cleanliness. The observation that the prevalence of *P. mirabilis* infections was at least partially nosocomial concurs with the study by Ray et al. [25], who emphasized the significant colonization risk posed by the pathogen in healthcare facilities where routine surveillance and sterilization can lack uniformity, particularly in the case of resource-poor facilities.

The study also found that comorbid diabetes is overly prevalent in infected patients, and this might indicate an immunocompromised disposition. Diabetes has previously been identified as a risk factor for various bacterial infections due to poor glycemic control, impaired neutrophil function, and microvascular complications. The literature, including the retrospective study by Albujassim et al. [27], corroborates this finding, noting that diabetic ICU patients are more prone to secondary bacteremia originating from urinary and respiratory sources. In contrast, hypertension and the absence of comorbidities were not strongly associated with infection risk in the present analysis. Hypertension appeared negatively associated with ICU stay duration in regression models, perhaps reflecting its better outpatient management and lower association with device dependence [32].

In terms of age and gender, the findings were less conclusive. While infected individuals were slightly older on average and age showed a modest positive relationship with ICU stay in all three regression models, the effect size was not pronounced [33]. This somewhat aligns with existing research that recognizes age as a general but not independently strong predictor of ICU infection risk once other clinical factors are controlled. Gender appeared balanced between groups, and although male gender retained a coefficient in the linear regression model, Lasso regression eliminated it, indicating that it lacks standalone predictive strength. These nuances suggest that demographic factors may not independently predict *P. mirabilis* infection as robustly as procedural exposures and comorbidities do [30].

An innovative component of this study is the application of predictive modeling to estimate ICU stay duration using linear, Ridge, and Lasso regression techniques. Notably, the Lasso model shrunk several coefficients (e.g., ventilator use, urinary catheter, *P. mirabilis* positivity) to zero, favoring model simplicity and variable selection over marginal predictive gains. Ridge regression, on the other hand, retained all variables with smaller effect sizes, reflecting its utility in handling multicollinearity. The linear model revealed high coefficients for central line use (3.24), hospital-acquired infection (2.46), and diabetes (1.05), pointing to their cumulative burden on ICU resources. The *P. mirabilis*, positive variable, had a negative coefficient (2.49) in the linear model, which may suggest that once patients are stabilized and treated, the infection itself does not prolong ICU stay as much as the complications or device-related factors associated with it [33].

Compared to the broader literature, this study stands out for its focused evaluation of *P. mirabilis* specifically, rather than grouping it under the generic category of “Gram-negative organisms.” This species-level analysis provides much-needed granularity, as called for by several recent reviews on ICU-acquired infections, including a meta-analysis by Muntean and Licker [31], which highlighted the underreporting of species-specific resistance patterns and clinical correlations. By isolating the impact of *P. mirabilis*, the study provides actionable insights for empirical therapy, infection prevention, and ICU staffing decisions.

Importantly, the study’s findings also reinforce the critical importance of antimicrobial stewardship in ICU settings. While resistance profiles were not deeply detailed in the results provided, previous literature has documented widespread resistance of *P. mirabilis* to third-generation cephalosporins and fluoroquinolones, with emerging resistance to carbapenems. In settings where empirical therapy precedes culture confirmation, misuse of broad-spectrum antibiotics can lead to poor outcomes and increased selection pressure. Thus, integrating local antibiograms and timely culture results into clinical workflows is an essential area also emphasized by studies in similar LMIC contexts [32].

In conclusion, this study validates and expands upon existing literature by confirming the central role of invasive devices, hospital-acquired infections, and diabetes in *P. mirabilis*-associated BSIs in ICU patients. While demographic variables such as age and gender play a lesser role, procedural and clinical factors are clear predictors of both infection risk and ICU stay length. Predictive modeling adds further value by identifying the most influential variables for ICU outcomes and illustrating the potential of data-driven decision-making in critical care. Future studies should include larger sample sizes, molecular resistance profiling, and prospective designs to validate and build upon these findings. Nonetheless, this study makes a timely and significant contribution to ICU infection management and microbial epidemiology.

The study’s retrospective design limited the ability to establish causal relationships. The sample size (n=30) was relatively small, which may restrict the generalizability of findings. Important variables such as AMR profiles, treatment regimens, and time to intervention were not captured, which could influence outcomes. Lastly, the study lacked molecular diagnostics, which could have provided deeper insights into strains, specific resistance, and transmission dynamics. Future studies should employ prospective multicenter designs with larger, more diverse ICU populations to validate findings and enhance generalizability. Incorporating molecular diagnostics and resistance gene profiling would clarify mechanisms driving *P. mirabilis *pathogenicity and resistance. Additionally, time, treatment metrics, antibiotic regimens, and clinical outcomes should be studied to evaluate therapeutic efficacy. Development of risk prediction models using machine learning could further aid in the early identification of high-risk patients. Lastly, intervention-based research focusing on infection control protocols, device management strategies, and antimicrobial stewardship programs will be essential to reducing BSI incidence and improving ICU outcomes.

## Conclusions

This study revealed that 53.3% of ICU patients had *P. mirabilis *BSIs, with a mean age of 59.6 years and an average ICU stay of 13.3 days. High rates of ventilator use (60%), central line placement (60%), and urinary catheterization (63.3%) were observed among infected patients. Hospital-acquired infections accounted for 56.7% of cases. Regression analysis identified central line use (β=3.24) and hospital-acquired infection (β=2.46) as key predictors of longer ICU stays. Diabetes also emerged as a significant comorbidity (β=1.05), reinforcing procedural and clinical vulnerabilities as major risk factors. By concentrating exclusively on *P. mirabilis* in ICU patients, this research fills an important knowledge gap in infection surveillance and hospital epidemiology. Collection and analysis of local epidemiological data, assessment of resistance patterns, and evaluation of patient and treatment-related risk factors associated with BSIs will help in refining infection control policies, optimizing empirical antibiotic use, and ultimately reducing infection-related morbidity and mortality in ICU.
